# Lessons learned from counting molecules: how to lure CENP-A into the kinetochore

**DOI:** 10.1098/rsob.140191

**Published:** 2014-12-10

**Authors:** Kristin C. Scott, Kerry S. Bloom

**Affiliations:** 1 Department of Molecular Genetics and Microbiology, Duke University, Durham, NC, USA; 2 Department of Biology, University of North Carolina at Chapel Hill, Chapel Hill, NC, USA

## 1. Introduction

Chromosome segregation requires the assembly of a multi-protein complex at the centromere, known as the kinetochore, along with re-organization of the cytoskeleton from an anastomosing microtubule network into a highly polarized bipolar spindle. Electron microscopy of chemically preserved kinetochore/microtubule attachment sites demonstrates a conserved trilaminar structure, from *Schizosaccharomyces pombe* to human [1–3]. Within this structure, the inner kinetochore associates with chromatin and the outer kinetochore forms the interaction surface for kinetochore microtubules. Although this ultrastructure has been known for years, and significant advancements been made in understanding the molecular, biochemical and functional properties of the over 65–90 conserved kinetochore proteins (yeast [4] and mammals [5]), the molecular architecture of the kinetochore/microtubule attachment site is largely unknown.

Kinetochores in budding yeast remain stable throughout the cell cycle, and during mitosis each associates with a single kinetochore microtubule, making it an ideal model to investigate the higher order structure of the inner kinetochore. During metaphase, the 16 sister kinetochores cluster and bi-orient between the two centrosomes or spindle pole bodies (SPBs), the microtubule organizing centre in yeast [6]. The sister kinetochore clusters are separated from one another by approximately 1 µm. The distance between sister kinetochores is remarkably conserved in yeasts, *Drosophila*, *Caenorhabditis elegans* and humans [7].

When *S. cerevisiae* kinetochore components are fluorescently tagged, individual proteins at single microtubule attachment sites cannot be resolved, however the cluster of 16 sister chromatids appears as a single diffraction-limited fluorescent signal [8]. The stereotypic organization of the yeast spindle allows one to investigate the number and spatial organization of kinetochore clusters in metaphase. The spindle can be visualized through fluorescent tagging of SPB components (e.g. Spc29-RFP), allowing quantitative measurement of the length of the spindle and geometrical position of the kinetochore. These spatial coordinates provide a reference, to which the *X* (parallel to the spindle) and *Y* (perpendicular to the spindle) coordinates of kinetochore proteins of interest can be mapped. Using this two-dimensional method, the linear (*X*) distance between the SPB and kinetochore proteins GFP-Cse4 and GFP-Ndc80 are comparable to measurements made using super-resolution microscopy [9,10]. In the *Y*-dimension, the outer kinetochore Ndc80 (Ndc80-GFP) is minimally displaced from the kinetochore microtubule plus-end (94 nm) consistent with its function in linking kinetochore microtubules to the inner kinetochore. The displacement of CENP-A (Cse4-GFP) is nearly twice that of GFP-Ndc80 (94 nm versus 181 nm) [9]. This finding is unaccounted in molecular models of the kinetochore that place a single CENP-A-containing nucleosome proximal to the kinetochore microtubule plus-end. Assuming an octameric structure, the CENP-A nucleosome in this model should be 5 × 11 nm, considerably smaller that the diameter of a microtubule (25 nm) and the observed displacement of the aggregate GFP-Cse4 signal from 16 kinetochores (181 nm).

**Figure 1.**
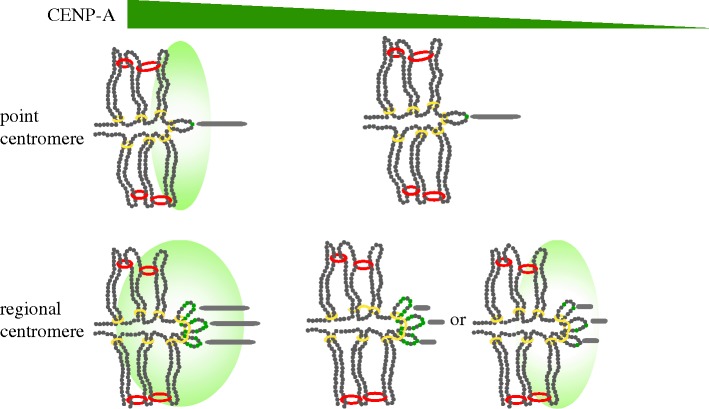
The inner kinetochore of point and regional centromeres. Centromeres are organized as a network of chromatin loops or folds with the foundational CENP-A-containing core chromatin (small green circles) adjacent to the kinetochore microtubule (grey bars). The pericentric region of a single chromatid (of 16 × 2 for replicated chromosomes) in one-half of the spindle is shown. The kinetochore (not shown) would connect the CENP-A-containing nucleosome (small green circle) to a single kinetochore microtubule in the point centromere and multiple microtubules in the regional centromere. Both point and regional centromeres contain a CENP-A cloud of accessory molecules (green shaded oval). The CENP-A cloud frames the cohesin barrel (red rings) organized around the pericentric chromatin in metaphase. Condensin rings (yellow) anchor chromatin structures at the centromere. Depletion of cellular levels of CENP-A/Cse4 in budding yeast (decreasing concentration denoted by green triangle, top) specifically affects the formation of the CENP-A/Cse4 cloud, and the fundamental inner kinetochore structure remains the same. Depletion of cellular CENP-A levels is tolerated in other organisms and may reduce the CENP-A abundance in the core, the cloud or both. Reduction of the cloud is indicated by loss of the large shaded oval. Reduction of the core (in the regional centromere) is denoted by fewer small green circles at the apex of the loops proximal to the multiple kinetochore microtubules (grey bars).

Model convolution of a mathematical simulation of the yeast spindle [9] provides the opportunity to model geometries that are consistent with experimental findings. Haase *et al*. [9] determined that the coordinates of Cse4-GFP were consistent with a single, CEN-positioned Cse4 nucleosome present at the inner kinetochore (and aligned with the outer kinetochore Ndc80 complex marking the microtubule plus-end) *and* a peripheral population of three to four Cse4 molecules per kinetochore associated with the chromosome surface. These results confirm and extend an earlier study demonstrating an average of five Cse4 molecules per kinetochore [11] and resolve prior uncertainty regarding the number and position of Cse4-containing nucleosomes in budding yeast [11–14]. Experimental findings indicate the peripheral population of Cse4 is confined to a ‘plate’ with a radius of approximately 250 nm perpendicular to the mitotic spindle (figure 1). This plate frames the cohesin barrel organized around the pericentric chromatin in metaphase [15]. Computer simulations predict that peripheral Cse4 is located at random positions (within 25 kb) flanking the well-positioned CEN nucleosome, variable among chromosomes and, consequently, below the level of current biochemical detection methods [11]. This result highlights the limitations of biochemical techniques in understanding higher order chromatin structure as the peripheral pool of Cse4 is not detectable by chromatin immunoprecipitation (ChIP) in wild-type cells [16,17]. These findings also advance our understanding of the budding yeast inner kinetochore during mitosis and define it as a chromatin surface, and structurally similar to the trilaminar arrangement observed at mitotic kinetochores of higher eukaryotes [1–3].

Realization of kinetochore protein geometry, and CENP-A in particular, reconciles protein counts attained from different microscopy platforms. Using fluorescence correlation spectroscopy (FCS) with its small cone of illumination focuses on the invariant (bright) pool, leaving the peripheral pool to background subtraction [18]. In wide-field microscopy, integrated intensity over a relatively large area (greater than 250 nm) leads to a larger estimate of protein counts than FCS-based methods [11,19]. Discussions regarding the nature of the GFP-constructs (internal versus C-terminal or N-terminal [14]) were tested in Lawrimore *et al.* [11] where different Cse4 fusion proteins yielded virtually identical protein counts (see [11, fig. 1]; Cse4-GFP B, C-terminal versus Cse4-GFP Cir^+^ and Cir^0^, internal). The evidence for functional diversification came from mutational experiments in which two pools could be separated genetically (via *pat1*Δ [9,20]). Loss of the peripheral pool was accompanied by a change from anisotropic to isotropic geometry of the clustered Cse4 molecules, as well as reduction in their number [9,20]. The extra molecules create a cloud of CENP-A that may be critical for centromere repair or neocentromere formation (figure 1, see below). Lando *et al*. [21] similarly noted that there may be an ‘elite’ core of CENP-A at the front-line of kinetochore microtubule engagement in fission yeast. Using super-resolution microscopy [14], Wisniewski *et al.* [14] confirmed the geometrical anisotropy of CENP-A in budding yeast. However without co-localization of the outer kinetochore components they were unable to define the position of the cloud relative to microtubule plus-ends.

How does dissection of the molecular architecture of the kinetochore in budding yeast inform our understanding of larger, regional centromeres? One point of consideration is the relationship between CENP-A-containing nucleosomes and microtubule attachment sites. The regional centromeres of the fission yeast, *Schizosaccharomyces pombe*, have an approximately 12–13 kb CENP-A-containing core chromatin domain that is constrained by chromatin barriers and flanked by domains of pericentromeric heterochromatin [22,23]. Like centromeres of budding yeast, the three *S. pombe* centromeres cluster throughout the cell cycle [24]. Recent examination of centromere clusters using quantitative photo-activated localization microscopy estimates the presence of approximately 15–35 molecules of CENP-A at each anaphase kinetochore, or an average of approximately 10–20 CENP-A-containing homotypic nucleosomes [21], similar to findings from Lawrimore *et al.* [11]. More intriguing is that although these budding and fission yeast species diverged 400–1000 Ma [25,26] and differ in centromere ‘type’ (point versus regional), the ratio between the number of CENP-A molecules and the number of microtubule attachment sites is consistent (budding yeast: 5–6 CENP-A/kMT; fission yeast: 25 CENP-A/4 kMTs = 6–7 CENP-A/kMT [11,21]).

Human centromeres are considerably larger than yeasts and it has been estimated that each kinetochore can interact with approximately 20–25 microtubules, though it is unclear how many load-bearing attachments occur at any given time. Several fluorescence-based microscopy studies of three-dimensional metaphase chromosomes have suggested that kinetochore size varies two- to threefold [27–29]. The plasticity of human kinetochores is further exemplified by studies demonstrating that the extent of CENP-A-containing core chromatin on stretched chromatin fibres is heterogeneous between homologous chromosomes and varies among non-homologous chromosomes and between individuals [30]. A recent study determined the number of CENP-A molecules present at kinetochores in human retinal pigment epithelium (RPE) cells [31]. Like the studies in yeast, Bodor [31] used microscopy to detect fluorescently labelled CENP-A. Using three independent quantification methods, the author found approximately 200 homotypic CENP-A nucleosomes at each mitotic kinetochore. Although these studies are limited to a single cell type, it is intriguing that the estimated ratio between CENP-A molecules and the number of microtubule attachment sites (400 CENP-A/20 kMTs = 20 CENP-A/kMT) is consistent with chicken (62 CENP-A/4 kMTs ∼ 15 CENP-A/kMT [32]) and within a factor of two or three of yeasts.

## 2. Centromere plasticity

Centromeres are frequently referred to as ‘plastic’ loci. This description originally referred to the fact that the primary sequence underlying the centromere varies within an organism and among many organisms; moreover, functional neocentromeres can assemble at various genomic loci. Likewise, the size of CENP-A-containing core chromatin domains varies within and among organisms. It has been known that the pool of CENP-A exceeds that required for accurate segregation function in humans [33]. Several recent studies extend and support this conclusion.

In *S. cerevisiae*, reduction of cellular Cse4 to approximately 50% of wild-type levels has a small, but measurable effect on segregation fidelity. In this study, Cse4 levels are indirectly reduced in a *pat1*Δ mutant [9] that exhibits 30× increase in chromosome loss [20]. It remains unclear whether the segregation defect is a direct consequence of the reduced level of cellular Cse4, or of the absence of Pat1, which has a demonstrated effect on kinetochore function [20]. Centromere chromatin is more accessible to DraI nuclease digestion and sister chromatid separation is delayed in *pat1*Δ mutants [20]. In *Candida albicans*, 33–50% of molecules in *rad51* or *rad52* mutants are tolerated [34], and segregation proceeds normally in *S. pombe* strains bearing a reduced amount of CENP-A at centromeres, although mild growth defects are observed [19,21]. Heterozygous RPE1 cells with a single integrated copy of CENP-A express about 50% of wild-type levels with a fractional increase (0.5–2.5%) in the appearance of cells with micronuclei. Thus, the critical quantity of CENP-A is approximately 100 CENP-A nucleosomes in humans, comparable to the size of the core fraction in budding yeast taking into account the number of kinetochore microtubules. In a separate study, centromere function was assessed following the conditional deletion of CENP-A. Intriguingly, functional centromeres were detected even after seven divisions in the absence of new CENP-A, suggesting that dilution to approximately 1% of the starting amount of CENP-A can be tolerated *in vivo* [35].

## 3. Core versus accessory/peripheral/cloud CENP-A molecules

A new aspect of centromere plasticity is the presence of CENP-A molecules outside of the core domain (CENP-A cloud, figure 1), and recent studies in the budding yeasts and chicken cells provide important insights into the functional significance of accessory CENP-A.

The nucleosomes in the *S. cerevisiae* pericentric region are dynamic [36], with the balance between eviction and insertion modulated by chromatin remodellers, including STH1/NPS1 and ISW2. Whereas the core Cse4 histone is stable in metaphase [14,37], its loss from a single chromosome would be catastrophic. The apparent confinement of peripheral Cse4 molecules to the vicinity of the kinetochore allows for rapid incorporation of Cse4 in the event of eviction at the centromere [9]. The proposal that a peripheral Cse4 is important for these rogue loss events is reminiscent of the abundance of Sir2 proteins at telomeres [38]. Gasser *et al.* [38] proposed a mechanism, known in enzymology as the Circe effect [39], in which a local ligand is enriched relative to the binding site. The Circe effect refers to ‘the utilization of attractive forces to lure a substrate into a site in which it undergoes a transformation of structure’ [39]. In situations such as budding yeast, where loss of a single nucleosome is catastrophic, it behoves the system to keep several molecules in the vicinity as a reservoir for contingencies.

Low levels of CENP-A have been detected adjacent to the core CENP-A domains in DT40 chicken cells. When a large portion of the Z centromere was conditionally deleted, neocentromeres most frequently formed near the original Z centromere [40]. Centromere proximal neocentromeres also assemble in *C. albicans*, following the conditional deletion of varying amounts of endogenous CEN1, 5 and 7. Peripheral or low levels of Cse4 are not detected biochemically outside of the defined centromere; however, based on studies of *S. cerevisiae*, it is reasonable to hypothesize that low levels at random positions would be undetectable by this method. Together, these studies suggest that peripheral CENP-A can preserve centromere integrity, either in *trans* by shifting the protein to where it is needed, or in *cis* by seeding a neocentromere.

**Figure 2.**
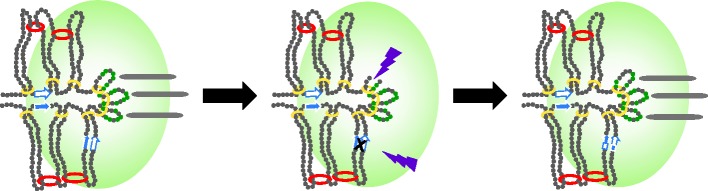
The CENP-A cloud and the Circe effect. The localization of the CENP-A/Cse4 cloud to the vicinity of the kinetochore may contribute to the centromere resilience. The CENP-A cloud (indicated by green shaded oval) represents the accumulation of CENP-A to the area, but not necessarily to the pericentric chromatin *per se*. If the CENP-A-containing core domain is damaged (purple bolts; loss of core CENP-A, small green circles, and breach of DNA), for example during replication stress, CENP-A molecules in the cloud may be quickly and efficiently re-incorporated by the recombination machinery (indicated by X, black) acting at centromere repeats (blue and white arrows). In the extreme event of centromere deletion, the core domain is compatible with neocentromere formation (not shown). A single strand of pericentric chromatin is shown in the half-spindle as described in figure 1.

*Candida albicans* has a regional centromere, but grows as a budding yeast. CENP-A appears as two clusters representing the aggregate of eight sister chromatids in metaphase of mitosis. As discussed above, the amount of CENP-A in wild-type is two times greater than that required for cellular viability [34]. CENP-A protein levels drop two- to threefold in *rad50* and *rad51* mutant cells [34]. RAD50 and RAD51 are required for homologous recombination and are essential in meiosis and when cells incur DNA damage, notably double-strand DNA breakage. It has been known for several years that replication forks pause when going through the centromere [34,41]. Mitra *et al.* [34] propose that the accessory pool of CENP-A may ameliorate potential damage from fork restart in DNA synthesis. Accumulation of single-stranded DNA at paused replication forks recruits protein involved in homologous recombination in the event of failed restarts or fork regression. Rad50 and/or Rad51 may bring in CENP-A for repair purposes. Interestingly, it has been shown that CENP-A is recruited to sites of DNA damage [42]. The recruitment of CENP-A to sites of damage may represent a conserved mechanism shared between the accessory pool at centromeres and sites of damage (figure 2).

In addition to DNA repair, there are reports that centromere proteins may function in recombination. Two members of the CCAN complex (CENP-S and CENP-X) that are proximal to chromatin were identified as MHF1 and MHF2, for their interaction with FANCM (Fanconi's anaemia complementation group M) [43]. It has been proposed that inter-repeat recombination is a mechanism to form loops [44]. Likewise, cohesion- and condensin-generated loops have been proposed as integral components of the spring-based mechanisms in centromere function [15,45–47]. A unifying mechanism for chromatin clamps (cohesion and condensin) and recombination may be loop formation, with the recombination function as a means towards this end in organisms with centromere repeats (figure 1).

## 4. Limits of malleability

Together, these recent studies suggest that functional centromeres are extremely malleable. Yet, aneuploidy, genome instability and some cancers can all be traced to defects in centromere structure and function. An outstanding question, then, is the cause(s) of the defects attributed to centromere dysfunction. Recent studies in fission yeast may provide some clues. The CENP-A-containing core domain is flanked by chromatin barriers, which prevent pericentric heterochromatin and centromeric cohesin from impinging on the CENP-A core [48]. The insertion of exogenous DNA into the barriers causes both structural and functional changes at the centromere. Cytologically, centromeres are decondensed, suggesting mislocalized cohesin and/or condensin. In addition, ChIP experiments demonstrate an increase in enrichment of CENP-A at the core domain in the cell population. These mutants have decreased viability and a high incidence of mitotic defects [49]. An intriguing hypothesis is that the higher order structure of the kinetochore, which may involve an intramolecular loop (or several loops, figure 1), is altered in these mutants, prohibiting or disrupting proper microtubule attachment. Although this remains to be experimentally tested, it is compelling that these mutant centromeres ‘cure’ themselves through an intra-centromere recombination-like mechanism that results in a precise excision of the exogenous DNA, restoration of wild-type levels of CENP-A and normal chromosome segregation.

## 5. Concluding remarks

Recent advances in quantitating the amount of CENP-A at endogenous centromeres have led to a molecular understanding of the inner kinetochore and have identified similarities among point and regional centromeres. The presence of a CENP-A cloud opens up new questions regarding mechanisms that lure proteins to active sites and poise cells for catastrophic events as suggested by W. Jencks several decades ago [39]. Future studies in yeast and other organisms will undoubtedly reveal additional information regarding the geometry/architecture/three-dimensional structure of the kinetochore and improve our understanding of the molecular defects that lead to missegregation/aneuploidy.
